# Stripe and spot selection in cusp patterning of mammalian molar formation

**DOI:** 10.1038/s41598-022-13539-w

**Published:** 2022-06-14

**Authors:** Wataru Morita, Naoki Morimoto, Keishi Otsu, Takashi Miura

**Affiliations:** 1 Department of Anthropology, National Museum of Nature and Science, Ibaraki, Japan; 2 Laboratory of Physical Anthropology, Department of Zoology, Graduate School of Science, Kyoto University, Kyoto, Japan; 3 Division of Developmental Biology and Regenerative Medicine, Department of Anatomy, Iwate Medical University, Iwate, Japan; 4 Department of Anatomy and Cell Biology, Graduate School of Medical Sciences, Kyushu University, Fukuoka, Japan

**Keywords:** Evolutionary developmental biology, Computational biology and bioinformatics

## Abstract

Tooth development is governed largely by epithelial–mesenchymal interactions and is mediated by numerous signaling pathways. This type of morphogenetic processes has been explained by reaction–diffusion systems, especially in the framework of a Turing model. Here we focus on morphological and developmental differences between upper and lower molars in mice by modeling 2D pattern formation in a Turing system. Stripe vs. spot patterns are the primary types of variation in a Turing model. We show that the complexity of the cusp cross-sections can distinguish between stripe vs. spot patterns, and mice have stripe-like upper and spot-like lower molar morphologies. Additionally, our computational modeling that incorporates empirical data on tooth germ growth traces the order of cusp formation and relative position of the cusps in upper and lower molars in mice. We further propose a hypothetical framework of developmental mechanism that could help us understand the evolution of the highly variable nature of mammalian molars associated with the acquisition of the hypocone and the increase of lophedness.

## Introduction

Phenotypic variation should be related to variation in developmental processes, ranging from epigenetic response to environmental stimuli to complex spontaneous pattern formation. Many genes and gene networks involved in developmental processes have been increasingly characterized^1,2^. However, it remains to be determined how gene networks and resulting interactions among signaling molecules elaborate complex pattern formation. In 1952, the British mathematician Alan Turing proposed a theory in which the interaction of two hypothetical molecules can serve as a system of spontaneous periodic pattern formation^3^. In this model, he assumed that two hypothetical molecules interact with each other and diffuse passively, whereby periodic patterns arise spontaneously. In higher dimensions, the system exhibits various interesting patterns depending on the reverse symmetry of the system^4,5^. This model was later rediscovered by Meinhardt and Gierer, and it was applied to many biological pattern formation systems^6^. The role of this model in the actual developmental processes has been controversial, but recent studies have shown that various developmental processes indeed utilize a similar mechanism, including animal coat markings^7^, feather buds distribution^8,9^, left–right asymmetry^10^, limb skeletogenesis^11^, and tooth morphogenesis^12–16^.

It has been proposed that a reaction–diffusion system can explain tooth morphogenesis at different levels. At a relatively global level, regulation of inter-molar size can be explained by an inhibitory cascade model. At a relatively local level, cusp formation within a single tooth can be explained by a patterning cascade model^17,18^. The individual tooth is considered to be highly self-regulated. The number and spatial patterning of cusps are determined by the iterative activation of secondary enamel knots that are epithelial signaling centers providing positional information, and by inductive interaction between the epithelium and the underlying mesenchymal cells^19,20^. The development of the tooth crown proceeds through various stages defined by the morphology of the epithelium (bud, cap, and bell stages). The transition from the bud to the cap stage is critical since morphological features are already determined at this stage. During the cap and bell stages, the size and shape of the tooth crown become apparent^21^. It has been proposed that this self-regulated process of cusp pattern formation is governed by the reiterative use of the same signaling pathways from a global to local level^20,22,23^.

While the molecular circuit that controls tooth morphology has been well studied, it remains unclear how exactly the variation in tooth morphology develops. One of the fundamental questions is unknown how differences between upper vs. lower molars (UM vs. LM) are specified during development. In many mammalian species, the UM and LM morphologies differ from each other^24^ even though they are assumed to be under the same genetic control and similar selective pressures. Mammals have acquired various evolutionary novelties that separate them from other vertebrates, including homeothermy, viviparity, pelage, auditory ossicles, and large brains; the diversification of tooth-type is another important feature^25,26^. In particular, the emergence of a multi-cusped tooth led to improved occlusal efficiency and the subsequent adaptive radiation^27^, which raises the question as to how each cusp is distributed in the tooth crown. In addition to the emergence of the multi-cusped tooth, mammal species evolved different morphology between upper and lower teeth.

In this study, we utilize the Turing model to elucidate the mechanism of cusp patterning in tooth morphogenesis. Specifically, we focus on differences between the UM and LM morphology in mice. In mice, the UM − LM difference is characterized by the difference in the number and relative position of the cusps, as in other mammal species. While the UM shows a clear cusp-cum-loph form where each cusp is connected by ridges, the cusps of LM are independent of each other. In addition, the inter-cusp ridges are less developed in LM than in UM. While tooth morphogenesis is a complex process, it can be modeled under the reaction–diffusion system. In two-dimensional (2D) pattern formation, the dynamics of the two morphogens yield either stripe or spot patterns^28^. The difference between UM and LM morphology may involve the generation of such a 2D structure that is repeated periodically in space. We thus hypothesized that the UM vs. LM difference in mice can be explained principally as stripe vs. spot patterns within the framework of a Turing system. In the case of tooth development, it has been proposed that bone morphogenetic proteins (BMP) act as activators, and fibroblast growth factors (FGF) and sonic hedgehog (SHH) act as inhibitors^12^.

Here, we test this hypothesis by taking two approaches. In the first “static” approach, we quantify the shape of the UM and LM using cross-sections at the middle height of the cusp. The second “developmentally dynamic” approach consists of constructing a computational Turing model to reproduce tooth development that corresponds to the morphogenetic stages. Specifically, the primary features of tooth development, such as the number and relative position of cusps, should be reproducible in models with parameter settings for stripe (UM) and spot (LM). While we use mice as a test model, this question is of special relevance for understanding the developmental mechanisms that produce the great diversity in molar morphology across a wide range of mammals.

## Results

### Morphometric analysis in fully formed teeth

**Figure 1:**
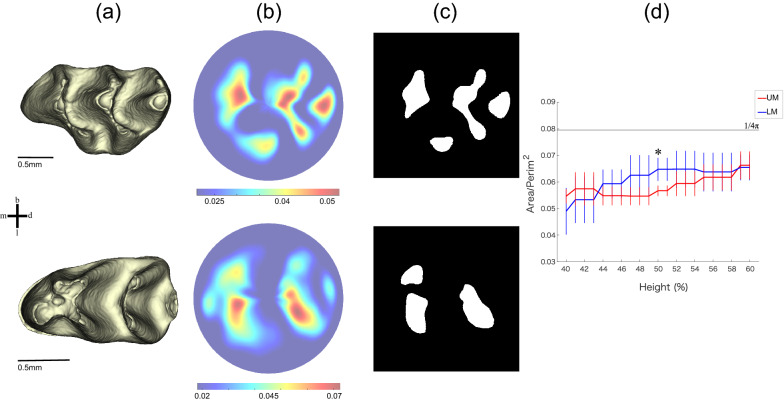
Scheme for 2D pattern extraction. (**a**) The 3D models of enamel–dentine junction. The three-dimensional surface models derived from µCT data were visualized by an in-house program, ForMATit, developed by N.M. [MATLAB-based (MathWorks, Version R2021b, https://www.mathworks.com/products/matlab.html)]. (**b**) Morphometric maps of the height from the cervical plane in UM and LM. (**c**) Binarized image at 50% of total height. White regions are above the threshold. Top and bottom panels show UM and LM, respectively. *m* mesial, *d* distal, *b* buccal, *l* lingual. (**d**) A dimensionless index calculated as area/squared perimeter is used to distinguish between stripe and spot patterns. Significant differences between UM and LM were detected (the number of samples, *n* = 10 for each of UM and LM; the probability of null hypothesis by Kruskal–Wallis test, p < 0.001) at 40–60% of the total height from the cervical plane. The mean and standard deviation at each threshold level are indicated with a polygonal line and whisker, respectively. The asterisk indicates the threshold at the 50% level where binarized images of (**d**) are derived. The black line indicates a constant in the case of circle (1/4π).

We reconstructed a 3D enamel–dentine junction (EDJ) model of each UM and LM mouse specimen, which we transformed into a circular 2D image using height from the cervical plane by a morphometric mapping method (Fig. 1). The difference between stripe and spot patterns was quantified by the average ratio of area to the squared perimeter for each object in a binary image. This stripe-like pattern should be smaller than that of the spot-like pattern, as the perimeter of each object in a stripe pattern is longer than that of the circular spotted pattern object. To detect a 2D pattern difference, we compared UM to LM while changing the threshold for binarization to within 40–60% of total cusp height. This pattern indicator for UM was significantly smaller than LM, suggesting that UM tends to have a stripe-like pattern, but LM has a spot-like cusp pattern.

### Mathematical analysis of mode doubling in a region growing system

**Figure 2:**
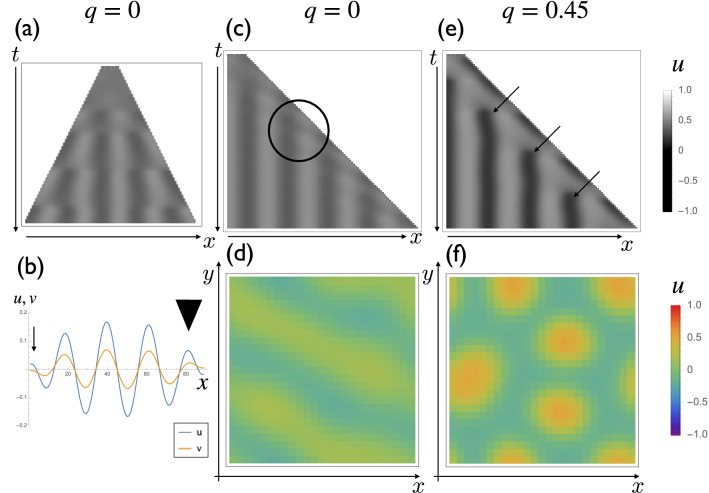
The mode doubling pattern in one dimensional simulations and stripe-spot selection in two dimensional simulations. (**a**) Pattern formation with a uniform domain growth with zero flux boundary conditions. The parameter set we used was: $f_{u} = 0.6,\;f_{v} = - 1,\;g_{u} = 1.5,\;g_{v} = - 2,\;d_{u} = 0.0002,\;d_{v} = 0.002,\;c = 1, \;q = 0$. We could observe the formation of new peaks restricted to the boundary. (**b**) The distribution of *u* (blue) and *v* (orange) at the timepoint of new peak formation. Splitting (arrowhead) and insertion (arrow) are observed simultaneously. (**c**) Pattern formation when growth is restricted to the right edge and the reaction term is symmetric ($q = 0$. Detailed explanations of *q* is in “Materials and methods” section). A new peak is always generated at the site of growth. It is not clear whether splitting or insertion occurs (circle). (**d**) Two dimensional pattern formation of the system (*q* = 0). The stripe pattern is formed. (**e**) Pattern formation with a nonzero *q* term (*q* = 0.45) when growth is restricted to the right edge. Insertion becomes predominant (arrows). (**f**) 2D pattern formation of the system with a nonzero *q* term (*q* = 0.45). The spot pattern is formed.

To understand the pattern formation mechanism, we utilize the Turing model, which is widely used to model tooth development^12^. Before generating complex 2D models, we utilized a simple 1D model with a growing domain to understand the pattern of mode doubling^29^. The pattern of mode doubling can be classified into three categories: insertion (formation of a de novo peak between two peaks), splitting (formation of new peaks by division of preexisting peak), and mode tripling (splitting and insertion take place simultaneously). At first, we tried uniform domain growth with zero flux boundary conditions. We observed that new peaks always are generated from both edges of the region, which differs from the way pattern formation occurs in vivo (Fig. 2a). This is because the whole system is symmetric, and a zero flux boundary condition can induce deviations from ideal patterns, which makes the region become susceptible to the collapse of the pattern induced by growth^29^. Insertion and splitting both occur at the edges (Fig. 2b).

We then introduced the asymmetry of growth patterns into the model. When only one side of the region grows, new peaks always are generated near the growing boundary (Fig. 2c). We use reaction terms that have reverse symmetry in this case. Consequently, it remains difficult to see whether a new pattern is generated due to the splitting of a preexisting peak or by the insertion of new peaks between two peaks. The 2D simulation of the same reaction term without domain growth results in a stripe pattern (Fig. 2d).

To represent the difference in UM and LM morphology, we introduce reverse asymmetry into the system by adding a quadratic term $q u^{2}$. It has been shown that the pattern formation process can be classified into splitting, insertion, and mode tripling depending on the reverse symmetry of the system^29^. We observed that the insertion pattern becomes dominant when a positive *q* term is introduced (Fig. 2e,f).

In all cases, the number of peaks is proportional to the domain size at a specific time. Therefore, we expect that the longer axis should have more peaks in a 2D system.

### Computational modeling of molar morphogenesis

**Figure 3:**
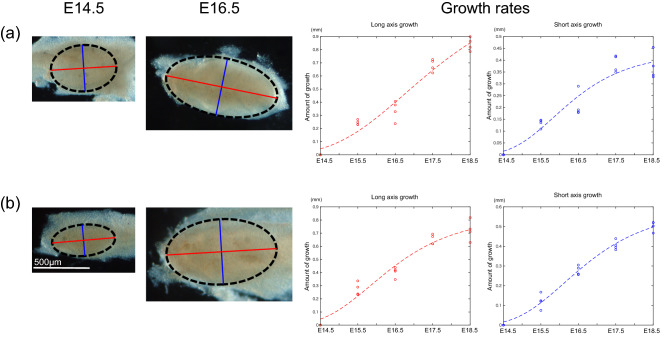
Modeling of molar growth. The length along the long and short axes of tooth germ is measured every other day from E14.5 to E18.5 (*n* = 4 for each time point) in UMs (**a**) and LMs (**b**). The parameters for fitting growth rates are the following: UM long axis, α = 1.267; β = 3.337; γ = 0.5316 (*r*^2^ = 0.96). UM short axis, α = 0.4295; β = 3.342; *c* = 0.9023 (*r*^2^ = 0.91). LM long axis, α = 0.8218; β = 2.895; γ = 0.7901 (*r*^2^ = 0.95). LM short axis, α = 0.5811; β = 3.577; γ = 0.7851 (*r*^2^ = 0.98). The ellipse with the black dashed line indicates tooth germ, and red and blue lines correspond to long and short axes, respectively.

To obtain data about how tooth germ size increases, we measured the length of the long and short tooth germ axes. We carefully isolated the UM and LM from the gnathic bones between embryonic day 14.5 (E14.5) and E18.5. During this period, the secondary enamel knots appear sequentially and regulate cusp patterning^30^. The long and short axes of the tooth germ were measured at various time points (number of samples, *n* = 4). To model the growth rates of tooth germs, growth was fit to a sigmoid curve [Eq. (1); Fig. 3].

We simulated spatial pattern formation of UM and LM development in mice as the concentration of the activator in a Turing model [Eq. (3)].

Our simulation was constructed under the following framework;

- The calculation of the elliptical domain is expanded by the growth function.
- Prepare two parameter settings that realize stripe and spot patterns.
- Morphogenesis of the UM and LM was simulated using parameter sets of stripe and spot patterns, respectively.

**Figure 4:**
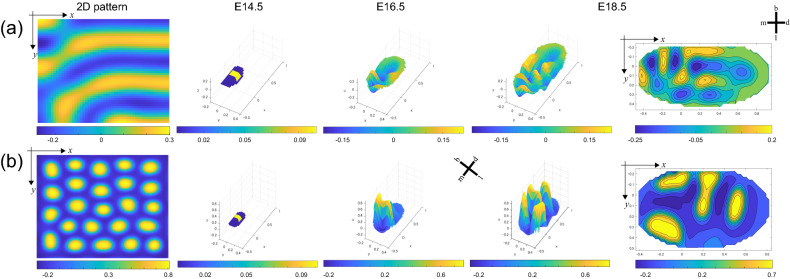
Simulation of molar morphogenesis. The parameters for UM that make stripe patterns are the following: (**a**) *f*_*u*_ = 0.4, *f*_*v*_ = − 1, *q* = 0, *c* = 1, *g*_*u*_ = 0.4, *g*_*v*_ = − 0.44, *d*_*u*_ = 0.006, *d*_*v*_ = 0.06. The parameters for LM that make spot patterns are the following: (**b**) *f*_*u*_ = 0.2, *f*_*v*_ = − 0.6, *q* = 0.45, *c* = 0.6, *g*_*u*_ = 0.2, *g*_*v*_ = − 0.32, *d*_*u*_ = 0.006, *d*_*v*_ = 0.06. The 2D images of stripe and spot patterns are formed after letting the system settle from an initially random configuration under the above parameter sets. Simulated concentration of activator (*u*) is represented in 3D at E 14.5, E 16.5, and E. 18.5. The final plain colormaps are also shown at E 18.5. The unit of *x* and *y* axis is in mm. *m* mesial, *d* distal, *b* buccal, *l* lingual.

As a starting point, the model includes the concentration of an activator that is located in the center of the domain. This initial condition corresponds to the primary enamel knot that appears in the cap stage of development (Fig. 4). The model for UM indicates that the first activator concentration appears on the mesiobuccal side, which then progresses in the mesiolingual and distal direction, where it finally results in three wave-like structures (S1 Video). In the LM simulation, the first activator concentration appeared on the mesial side and then shifted toward the lingual direction (S2 Video). Another concentration then emerged on the distal side and split from the lingual to the buccal side, at which point the final concentration was added distally. These simulations of molar morphogenesis are consistent with earlier reports that described the order of molar emergence in UM^31^ and LM^22,32^, respectively. The Turing model involved in a region growing system based on empirical data showed that UM and LM could be described by a parameter set that yields stripe and spot patterns, respectively. Our model successfully reproduces both UM and LM cusp patterns, as well as the actual process of tooth development.

## Discussion

In this study, we tested the hypothesis that differences in UM and LM morphologies are explained by stripe and spot patterns using mice as a model. First, we tested the hypothesis with a “static” analysis of tooth shape. Results show that UM exhibits smaller index values (area/perimeter), which indicates a more complicated shape pattern compared to LM. Second, we tested the hypothesis with a “developmentally dynamic” analysis using the Turing model as a framework. The model revealed that the parameter settings for stripe vs. spot patterns matched the UM vs. LM morphologies. Furthermore, our tooth model reproduced the relative location of cusps as well as the sequence of cusp formation in UM and LM. Thus, both analyses supported the hypothesis that UM vs. LM morphologies have developed via stripe and spot patterns.

Turing patterns with domain growth have not been well studied until recently because the growth of material is not a common phenomenon in physical or chemical systems. In 1995, Kondo and Asai^7^ described that the stripe pattern in angelfish could change due to the growth of the fish, which invoked interest in pattern formation in growing domains. Pattern formation in a growing domain has been analyzed theoretically in one-dimensional systems^29^. In particular, they begin by numerically implementing the Turing model on a growing domain and observe the increase of activator peaks due to the domain growth, which is called mode doubling. The steady-state solution of the Turing pattern on a growing domain was analyzed using a piecewise linear approximation of the reaction term, and the condition for the distinction of these three dynamics turned out to be correlated to the reverse symmetry of the system^29^. Intriguingly the reverse symmetry of the system also is important for stripe–spot selection (in this study, we use the term ‘selection’ to refer to the choice between stripe and spot patterns in the context of the Turing system but not to natural selection in evolutionary theory)^4^. We showed the relationship between stripe–spot selection and mode doubling (Fig. 2), which has not been fully elucidated analytically.

Previous studies have simulated tooth development using a morphodynamic mechanism in which inductive and morphogenetic mechanisms interact dynamically with each other^12,33,34^. While tooth development has been considered highly self-regulated, a recent study showed that external factors, such as physical interaction with the jawbone, is relevant for the regulation of tooth morphogenesis^35^. In this study, we simplified the model by not considering this potential external factor. Our model is implemented by two independent mechanisms: (a) global size regulation based on empirical data, and (b) local cusp patterning based on a Turing model. We thus call it a semi-morphodynamic model. While the relative position of the cusps is altered moderately, the sequence of cusp formation remains essentially unaffected with or without the external factor. While our model does not allow us to evaluate the effects of external physical constraints, it does allow us to examine the reciprocal relationship between growth and the Turing pattern.

We tested how the overall size regulation of the tooth germ affected the morphogenesis of EDJ. The swapping experiments in silico between the growth function in the UM and the reaction–diffusion parameter set for LM (and vice versa) show that the spatiotemporal cusp patterns are not changed drastically (S3 Video and S4 Video). One implication of this swapping simulation is that tooth morphogenesis might be more sensitive to local 2D patterning than to global size regulation. This indicates that the increased body size and associated change of the growth pattern along the course of evolution have relatively minor effects on the pattern of cusp formation. This is consistent with the notion that rats and mice exhibit similar tooth morphologies despite more than two-fold differences in body mass. It should be noted, however, that slight modifications can occur with such swapping. For example, the UM model that grew by LM growth rates yielded additional distal cusps, which might also be consistent with the patterning cascade model, where variation in small-sized cusps should be cumulative, and random variation should appear easily in later-developing cusps^36^.

The related question is to identify the morphogens that act in the system described here. Actual candidates for activators and inhibitors of Turing systems have been proposed in various biological systems. In most cases, the molecules that fall into some of the most important categories of the signaling pathway for development, such as fibroblast growth factors (FGF), bone morphogenetic proteins (BMP), sonic hedgehog (SHH), and Wnt^37^, correspond to it. As we mentioned previously, in the case of tooth development, it has been proposed that BMPs act as activators, and FGF and SHH act as inhibitors^12^. These proteins also should be relevant for determining the morphology of EDJ. It remains unknown, however, exactly how each potential morphogen is regulated during the EDJ morphogenesis. Our model consisted of only two morphogens, which is clearly an oversimplification. Thus, it is difficult to apply this hypothetical model directly to certain molecules that function during real odontogenesis. Our simple model only describes the phenomenon of interest, but it may provide additional insight into morphological evolution, and it provides a better understanding of underlying developmental mechanisms^38,39^.

We may extend the framework presented here to human molars. Fig. S1 shows the EDJ of the first UM and LM of humans. The sequence of cusp formation is shared in both UM and LM in humans: mesiobuccal → mesiolingual → distobuccal → distolingual^40^. The UMs of humans exhibit a ridge, called the oblique crest, which connects the mesiolingual and distobuccal cusps. The grooves that separate these cusps are only weakly expressed or not present at all. In contrast, the LMs of humans have cusps that are delimited clearly from each other by deep grooves. Given these morphological differences between UM and LM in humans, we suggest that stripe–spot selection could also apply to human molars. While this hypothesis remains to be tested, our computational model could provide new insights into the morphogenesis of human molars. For example, recent studies on genetic disorders, such as ectodermal dysplasia, have identified genes involved in the regulation of cusp number and shape^41,42^. Currently, it is unknown whether tooth malformation is associated with changes in the sequence of cusp formation and/or it is related to the conversion between stripe- and spot-like patterns of cusp formation. Most genetic studies in mice, however, have shown reductions in the size and shape of teeth, and it is difficult to increase tooth complexity without modifying multiple signaling pathways^14,43^. However, changes in a particular signaling molecule, such as overexpression of *Edar*, can result in an increased number of spiky cusps^44^, which might be correlated with parameter changes in the Turing model. Thus, simulation-aided approaches have the potential to link experimental studies using model species, such as mice, with clinical research in humans, which might aid in the prevention and treatment of tooth malformation.

Our modeling suggests the difference between stripe-like UM and spot-like LM in cusp patterning. We hypothesize that stripe vs. spot patterning holds as a general rule for the evolution of mammalian teeth. For example, consider the proboscidean molar morphology. Between the Miocene and Holocene periods, the molars of ancestral ‘gomphotheres’ possessed spot-like conical cusps arranged in transverse rows on the crown, while recent Elephantidae exhibited more stripe-like incised ridges for shearing and cutting^45^. Thus, it is possible to detect evolutionary changes in molar morphology change utilizing 2D patterning in a Turing model, as implemented in this study. This hypothesis, however, should be tested using phylogenetic analyses based on fossils, computational modeling, and experimental reproduction from spot-like to stripe-like teeth.

**Figure 5:**
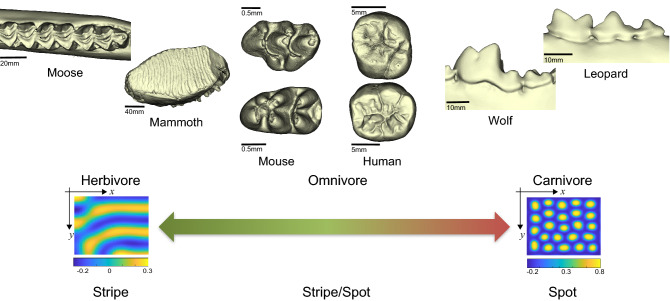
The proposed relationship between patterning and dietary habits. Tooth images of mammoth, moose, wolf, and leopard were obtained from MorphoSource.org (Media IDs: 8705, 8288, 7773, and 7779, respectively). The three-dimensional surface models derived from µCT data were visualized by an in-house program, ForMATit, developed by N.M. [MATLAB-based (MathWorks, Version R2021b, https://www.mathworks.com/products/matlab.html)].

Distinct pattern formation may be associated with dietary habits. Figure 5 shows that herbivores tend to have stripe-like teeth that are characterized by well-developed intercusp ridges, including the lophodont, loxodont, and selenodont. On the other hand, carnivores have spot-like cuspidate teeth, such as the carnassial (secodont) or denticulate. Omnivores are in between these two extremes. Although some omnivorous species may have different patterns between their UM and LM, such as mice (and perhaps humans), a diverse evolutionary pattern appears across mammals that roughly corresponds to their dietary adaptations.

Several characteristics of dentition are of special relevance for highly diversified mammal dietary habitats, such as relative molar size^18^ and crown surface complexity^46,47^. It has been proposed that the taxonomic diversification of mammals was associated with the gain of herbivory, which subsequently led to changes in molar tooth morphology. Specifically, the increase in the number of lophs led to the “complexity” of tooth morphology that resulted in the ability to consume fibrous plant foods^48^. Such “complexity” includes high-crowned teeth with multiple lophs and suggests that this resulted in the loss of intermediate crown types. We propose that an increase in lophedness through evolution could correspond to the switch from spots to stripes in cusp patterning. Along with such a switch of the developmental program, the acquisition of a hypocone could have played a major role. Hypocones are considered to be a key innovation associated with the taxonomic diversification of herbivorous mammals^27^. In the framework of our Turing model, this change is associated with mode doubling.

The acquisition of the hypocone has evolved more than 20 times convergently among various lineages even though there are several options for adding one cusp during odonotogenesis^27^. In terms of mode doubling, the subsequent cusp appears in mode tripling under the stripe pattern cusp formation, which suggests that the hypocone would not necessarily be derived from a certain cusp or structure, but it was acquired independently in the various species in a various approach^15,27,49–52^. Our hypothesis of switching from spot to stripe in cusp patterning is consistent with an increase in lophedness and the adaptive radiation of mammals with hypocones during the Cenozoic era.

Although the UM and LM of an individual are under identical genetic control with a common developmental architecture, most mammals have distinct morphological patterns, which suggests that the algorithm applied to UM and LM is different. Our computational modeling implies that final molar morphology could be linked to 2D pattern formation attained only by slight changes in model parameters. This suggests that the evolution of disparate morphologies may not require extensive modification of the developmental process and permits the diversification of molar morphology in mammals.

## Materials and methods

### Micro-CT data and 3D reconstructions

Unworn molars were obtained from the ICR mice. The first UM and LM were extracted at a postnatal age of two weeks. A total of 20 molars (*n* = 10 for each of UM and LM) were scanned using a µCT scanner (ELE SCAN, Nittetsu Elex, Japan; housed at Niigata University) with the following parameters: tube voltage: 72 kV and tube current: 11 µA. This resulted in an isotropic voxel resolution of 5 µm. Since the enamel–dentine junction (EDJ) in the fully formed tooth can be used as a proxy for the final configuration of the inner enamel epithelium that resulted from the patterning phase of development^53^, we selectively reconstructed EDJ for the analysis. Tooth segmentations were made between enamel and dentine, and reconstruction of the 3D model was performed with Amira (FEI Visualization Science Group).

### 2D pattern extraction and analyses

We evaluated the difference in cusp shape between UM and LM by extracting their 2D patterns. To normalize the different shapes of UM and LM, we projected the 3D surface to a normalized circular image. A 3D surface model of EDJ is transformed into a 2D circular image by means of morphometric mapping^54,55^. Before mapping, each tooth was aligned horizontally at its cervical line and centered using the centroid of the cervical line. The height from the cervical plane was used as a morphometric dataset for the analysis, which was sampled over the entire EDJ surface. To extract the 2D patterning, the morphometric map was binarized by using different values of thresholds. We used thresholds ranging from 40 to 60% of total EDJ height from the cervical plane because this threshold should be sensitive to pattern identification. For example, higher thresholds would only represent information on cusp tips, while lower thresholds represent the shape of the basement. Using the binarized image, we then calculated a dimensionless index of the area divided by the squared perimeter for each object, which allowed us to detect the 2D pattern difference between stripes and spots. If the pattern is spot-like, this variable should be close to that of a circle (1/4π). On the other hand, the stripe-like pattern should have smaller values because each object exhibits a complicated shape whose perimeter is relatively larger than its area.

### Measurements of tooth germ growth rates

All animal experiments were conducted in compliance with ARRIVE guidelines. The protocol for experimentation was approved by the Institutional Animal Care and Use Committee (Approval no. 27-044) of Iwate Medical University, and all methods were performed in accordance with relevant guidelines and regulations. The development of UM and LM was observed from embryonic day 14.5 (E14.5), when it starts the invagination of the epithelium and condensing mesenchyme to form its cap, until stage E18.5, when the crown cusp pattern is settled^21^. Although most cusps develop during the early bell stage, tooth mineralization has not begun. At noon of the day, when the vaginal plug was observed was considered E0.5. The UM and LM were excised from ddy mice (Japan SLC, Shizuoka, Japan) and fixed in 4% paraformaldehyde. Assuming the tooth germ is an ellipse, the long and short axis was measured every other day (Fig. 3). The Gompertz double exponential model was fitted to mean values of growth along the long and short axis from E14.5 as follows:

1 $$f(t) = \alpha e^{{ - \beta e^{ - \gamma t} }}$$

where α is the upper limit, β defines the value at *t* equal to zero in conjunction with α, and γ indicates the inclination.

### Numerical simulation of Turing pattern in a 1D growing region

**Figure 6:**
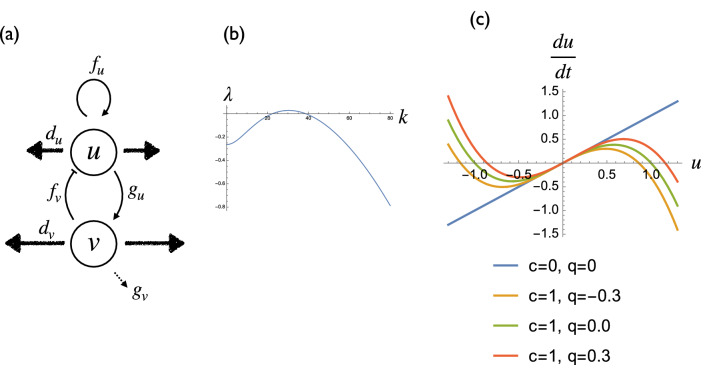
Definitions of the model parameters. (**a**) Schematic representation of the six linear parameters of the model. (**b**) Dispersion relation of the Eq. (2) ($f_{u} = 0.6,\;f_{v} = - \,1,\;g_{u} = 1.5,\;g_{v} = - \,2,\;d_{u} = 0.0002,\;d_{v} = 0.002$). (**c**) Effect of two nonlinear terms *c* and *q* on the *u* dynamics.

One-dimensional mockup models were implemented using *Mathematica*. The reaction–diffusion equations were a modified FitzHugh–Nagumo system^56,57^ to allow the mathematically simple switch between stripe and spot patterning with quadratic and cubic terms^28^. The equations are as follows:

2 $$\begin{aligned} \frac{\partial u}{{\partial t}} &= f_{u} u + f_{v} v + qu^{2} - cu^{3} + d_{u} \Delta u \\ \frac{\partial v}{{\partial t}} &= g_{u} u + g_{v} v + d_{v} \Delta v \end{aligned}$$

where *u* and *v* are standardized activator and inhibitor concentrations, respectively. The meanings of the linear parameters are described in Fig. 6a. *f*_*u*_ represents the positive feedback of the activator. *f*_*v*_ represents the inhibition of activator production by the inhibitor. *g*_*u*_ represents the promotion of inhibitor production by the activator. *g*_*v*_ represents the decay of the inhibitor. *d*_*u*_ and *d*_*v*_ are the diffusion coefficients of the activator and the inhibitor, respectively.

We confirmed that the parameter set gives vault-like dispersion relation $\lambda (k)$ (Fig. 6b), indicating that the system has the pattern formation capacity. *c* represents the saturation of the activator. Since the existence of positive feedback, activator concentration tends to go to infinity ($c = 0$ in Fig. 6c). Therefore, we set the upper and lower limit of the activator concentration using this term ($c = 1$ in Fig. 6c). *q* represents the asymmetry of the *u* reaction term. When *q* is positive, the equilibrium point is closer to the lower limit than the upper limit. This corresponds to the lower shift of the equilibrium point, which can be implemented by several situations:

1. Constant removal of the activator (for example, by an extracellular molecule that captures the activator).
2. Additional expression of pseudoreceptor of the activator.

Similarly, when *q* is negative, the equilibrium point is closer to the upper limit than the lower limit. This corresponds to the upper shift of the equilibrium point. There are several biologically plausible situations to implement this effect.

1. Constant addition of the activator from an external source (for example, from adjacent tissue).
2. Additional expression of constitutive active receptor of the activator.

Both reaction and diffusion terms are implemented using an explicit Euler scheme. To implement uniform domain growth, we increased the number of lattices at regular intervals. Concentration values of increase were calculated by linear interpolation as follows:

3 $$\begin{aligned} u(i,\;t + \Delta t) &= \frac{n + 1 - i}{n}u(i,\;t) + \frac{i - 1}{n}u(i + 1,\;t)\\ u(1,\; t + \Delta t) &= u(1,\;t)\\ u(n + 1,\; t + \Delta t) &= u(n,\;t) \end{aligned}$$

where *n* is the number of lattices at time *t*.

### Modeling pattern formation of molar morphology

The model combines growth rates of tooth germ inferred from empirical data and reaction–diffusion parameters to simulate cusp patterning. Spatial cusp pattern formation for UM and LM in wild-type mice was simulated with a system of reaction–diffusion equations [Eq. (2)] in a growing elliptic domain. The numerical simulations were undertaken with Dirichlet (i.e., fixed) boundary conditions. The growth was implemented as a growing ellipse that expanded into four directions: mesial, distal, buccal, and lingual. Given the tooth shape in adult mice, we assumed growth along the disto-lingual direction was greater than that along the mesio-buccal direction, setting the growth rate along the disto-lingual direction four times higher than along the mesio-buccal direction. We assumed *u* and *v* of the added lattices in UM to be 0 and − 0.0012, respectively, and both were 0 in LM. Under these initial conditions, the activator concentration was located at the center of the elliptic domain, which corresponded to the primary enamel knot that expresses during the cap stage of development.

## Supplementary Information

Supplementary Figure S1.

Supplementary Video S1.

Supplementary Video S2.

Supplementary Video S3.

Supplementary Video S4.

Supplementary Legends.

## Data Availability

Correspondence and requests for materials should be addressed to W.M.
