# Supplementary figures and images for: Stripe and spot selection in cusp patterning of mammalian molar formation
Source: Sci Rep. 2022 Jun 14;12:9149. doi: 10.1038/s41598-022-13539-w (PMC9197828; doi:10.1038/s41598-022-13539-w)

a

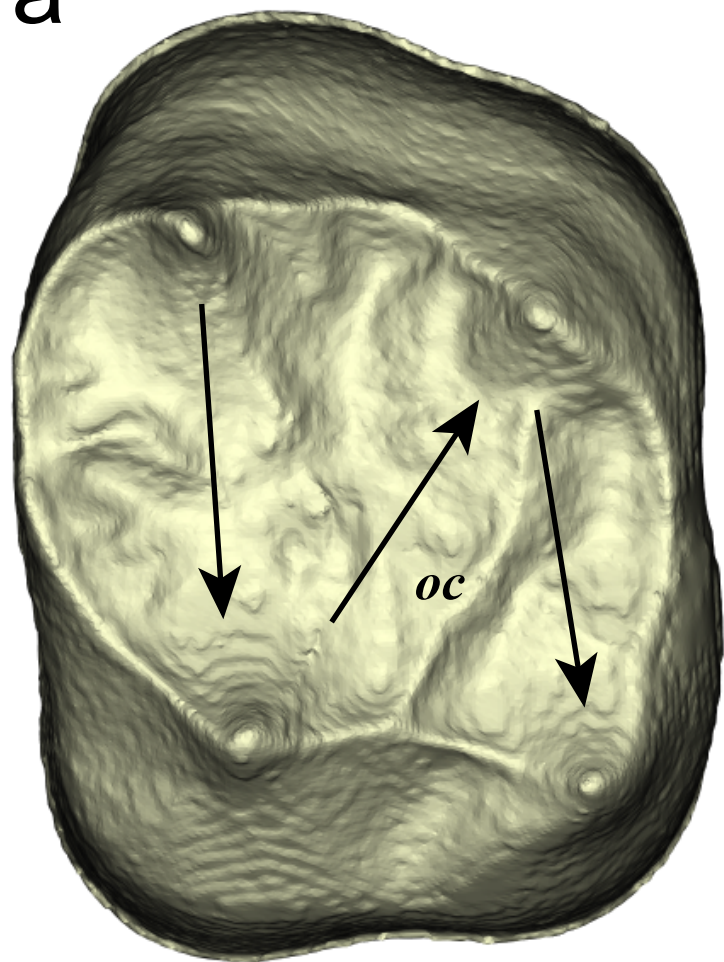

10mm

b

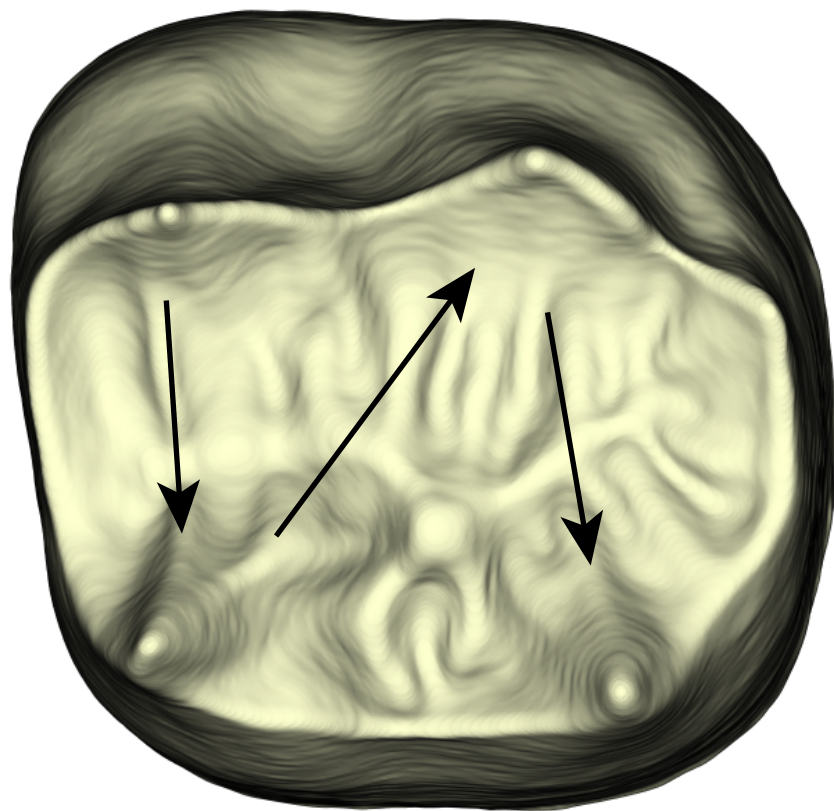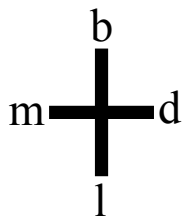

Supplement: Supplementary file 1 — Supplementary Figure S1. [file 41598_2022_13539_MOESM1_ESM.pdf]
