# Supplementary material for: Stripe and spot selection in cusp patterning of mammalian molar formation
Source: Sci Rep. 2022 Jun 14;12:9149. doi: 10.1038/s41598-022-13539-w (PMC9197828; doi:10.1038/s41598-022-13539-w)
Supplement: Supplementary file 6 — Supplementary Legends. [file 41598_2022_13539_MOESM6_ESM.docx]

**Supplementary material**

**S1 Video.** Simulation of UM. The unit of x and y axis is in mm and the concentration of activator (u) is represented along the third dimension.

**S2 Video.** Simulation of LM. The unit of x and y axis is in mm and the concentration of activator (u) is represented along the third dimension.

**S3 Video.** Simulation of UM in the LM growth rates. The unit of x and y axis is in mm and the concentration of activator (u) is represented along the third dimension.

**S4 Video.** Simulation of LM in the UM growth rates. The unit of x and y axis is in mm and the concentration of activator (u) is represented along the third dimension.

**S1 Fig.** The three-dimensional model of enamel–dentine junction of human UM (a) and LM (b). The three-dimensional surface models derived from µCT data were visualized by an in-house program, ForMATit, developed by N.M. [MATLAB-based (MathWorks, Version R2021b, https://www.mathworks.com/products/matlab.html)]. Arrows represent the order of cusp formation during development. *oc*: oblique crest, m: mesial, d: distal, b: buccal, l: lingual.
